# Comparison of Two Intraocular Lens Implantation Techniques in Pediatric Cataract Surgery in Terms of Postoperative Complications

**DOI:** 10.4274/balkanmedj.2017.1504

**Published:** 2018-03-15

**Authors:** Mustafa Erdoğan Cicik, Cezmi Doğan, Selim Bölükbaşı, Mehmet Necdet Cinhüseyinoğlu, Osman Şevki Arslan

**Affiliations:** 1 Department of Ophthalmology, İstanbul University, Cerrahpaşa School of Medicine, İstanbul, Turkey; 2 Clinic of Ophthalmology, İstanbul Okmeydanı Training and Research Hospital, İstanbul, Turkey; 3 Clinic of Ophthalmology, Private Birinci Eye Hospital, İstanbul, Turkey

**Keywords:** Cataract, intraocular lens, posterior capsule opacification, posterior capsulorhexis

## Abstract

**Background:**

Pediatric cataract surgery differs substantially from adult cataract surgery. Numerous studies have focused on reducing the development of postoperative complications.

**Aims:**

To compare two intraocular lens implantation techniques used in pediatric cataract surgery in terms of postoperative complications.

**Study Design:**

Case-control study.

**Methods:**

Patients who underwent pediatric cataract surgery and intraocular lens implantation between 2008 and 2016 were evaluated in this retrospective study. Patients aged 3-15 years with unilateral or bilateral cataract and without corneal pathology were included in the study. The patients were categorized into the following two groups: those who underwent posterior capsulorhexis and anterior vitrectomy using in-the-bag intraocular lens implantation (group 1) and those who underwent posterior capsulorhexis and intraocular lens implantation with the optic fixed behind the posterior capsulorhexis (group 2). Rates of postoperative visual axis opacification and complications (glaucoma, posterior synechiae, uveitic reaction, and intraocular lens decentration) were evaluated in these groups. The implanted intraocular lenses were either monoblock (AcrySof SN60AT intraocular lens), triple-piece (AcrySof MA60BM intraocular lens) foldable hydrophobic acrylic lenses, or multifocal lenses (AcrySof IQ ReSTOR).

**Results:**

This retrospective study included 52 eyes of 37 patients. Group 1 comprised 26 eyes of 20 patients and group 2 comprised 26 eyes of 17 patients. During the follow-up, visual axis opacification was observed in two patients in group 1 but no patients in group 2. Regarding postoperative complications, there was no uveitic reaction, posterior synechiae, or intraocular lens decentration in either group. There was no significant difference between the groups in terms of the postoperative complications (p>0.05).

**Conclusion:**

There was no significant difference between in-the-bag intraocular lens implantation after posterior capsulorhexis and intraocular lens optic capture through posterior capsulorhexis in terms of the complications.

Congenital cataracts are the most common preventable cause of blindness in children (1,2). Correcting congenital cataracts in children presents different challenges than those of adult cataract surgery, primarily due to the fact that children’s eyes have a shorter axial length, steeper corneal curvature, and more elastic capsule compared to those of adults. The most important factor affecting postoperative visual acuity in cataract surgery is the development of posterior capsule opacification (PCO), which leads to decreased vision and amblyopia. While PCO is treated by yttrium-aluminum-garnet laser capsulotomy in adults, this procedure is difficult to perform on children and requires repeated surgical interventions. Therefore, posterior capsulorhexis is performed to prevent postoperative PCO. The reported rate of PCO development in children when posterior capsulorhexis is not performed during cataract surgery is 100% (3,4). 

In addition to posterior capsulorhexis, various other surgical approaches have been employed in cataract surgery to prevent PCO. Primary anterior capsulorhexis, posterior capsulorhexis, and anterior vitrectomy are routinely performed if an intraocular lens (IOL) will not be implanted. If IOL implantation is planned, the IOL can be placed in the sulcus and stabilized by capturing the IOL optic behind the anterior capsulorhexis (haptic in the sulcus, optic behind the anterior capsulorhexis). Alternatively, the IOL can be placed in the bag (optic and haptic in the bag), or the optic component can be captured behind the posterior capsulorhexis while placing the IOL haptic in the bag (haptic in the bag, optic behind the posterior capsulorhexis) or in the sulcus (haptic in the sulcus, optic behind the posterior capsulorhexis). However, the close contact between the iris and the haptic in this method is undesirable. The aim of this study was to compare the rates of visual axis opacification (VAO) and postoperative complications (uveitic reaction, posterior synechiae, glaucoma, and IOL decentration) in pediatric cataract patients who have undergone IOL implantation in the capsular bag after posterior capsulorhexis and anterior vitrectomy with those of patients who have undergone posterior capsulorhexis and IOL implantation with the haptic in the bag and the optic positioned behind the posterior capsulorhexis (optic capture).

## MATERIALS AND METHODS

This study was approved by the local ethics committee and was conducted in accordance with the principles of the Declaration of Helsinki. Pediatric patients who underwent cataract surgery and IOL implantation between 2008 and 2016 were evaluated in this retrospective study. Patients aged 3-15 years with unilateral or bilateral cataract and without any corneal pathology were included in the study. Patients with other systemic and ocular pathologies were excluded. Patients were divided into two groups, one group consisting of patients who underwent posterior capsulorhexis and anterior vitrectomy with the IOL implanted entirely in the capsular bag (group 1) and the other consisting of those who underwent posterior capsulorhexis with IOL optic capture through the posterior capsulorhexis (group 2). Rates of postoperative VAO and complications (glaucoma, posterior synechiae, uveitic reaction, and IOL decentration) were evaluated in these groups. IOL master and Lenstar were used to calculate the IOL power. In cases where measurements could not be made using these devices, A-scan ultrasonography was used and keratometry values were calculated using an autorefractor keratometer. Age-appropriate keratometry values were used for patients for whom keratometry could not be performed. The Sanders-Retzlaff-Kraff formula was used to determine the IOL power, which was calculated as 10% lower than the emmetropic value for patients aged between 3 and 6 years and as emmetropic for those aged 7 years and older. All implanted IOLs were monoblock (AcrySof SN60AT IOL), triple-piece (AcrySof MA60BM IOL) foldable hydrophobic acrylic lenses, or multifocal lenses (AcrySof IQ ReSTOR).

### Surgical technique

All surgeries were performed under general anesthesia by the same two surgeons. Pupils were dilated preoperatively using tropicamide 1%, cycloplegin 1%, and phenylephrine 2.5%. In patients whose pupil width was not sufficient, adrenaline was administered to the anterior chamber to achieve further dilation. Side ports were created in the superior nasal and temporal regions, and the anterior chamber was filled with a dispersive viscoelastic. The anterior capsule was stained with trypan blue under air for better visibility when necessary. A 2.4 mm primary incision was made at the 12 o’clock position. After creating a flap in the anterior capsule using a 27-gauge cystotome, an anterior capsulorhexis with a diameter of approximately 5 mm was made using microcoaxial forceps. Following hydrodissection, the lens material was removed by bimanual irrigation and aspiration. The capsular bag was filled with a cohesive viscoelastic, and a tear was created in the posterior capsule using a 27-gauge cystotome while administering the dispersive viscoelastic into Berger’s space, after which a posterior capsulorhexis was created using microcoaxial forceps. In group 1 patients, triamcinolone-assisted anterior vitrectomy was performed after this procedure and the foldable IOL was placed in the bag between the cohesive viscoelastic-filled anterior and posterior capsule. In group 2 patients, the foldable IOL was placed in the bag after performing posterior capsulorhexis and the optic was captured behind the capsulorhexis, which had a slightly smaller diameter (3-4 mm) than that of the IOL optic. After removing the viscoelastic by aspiration, the side ports were closed by stromal hydration and tested for leakage using a sponge. When the anterior chamber could not be stabilized, the incisions were closed using 10/0 sutures. The operation was completed with a subconjunctival injection of gentamicin and dexamethasone. Postoperatively, the patients were started on topical dexamethasone and moxifloxacin drops at an initial dose of eight times daily, which was gradually decreased and discontinued within 4-6 weeks. Tropicamide 1% was administered as a mydriatic agent. The patients were examined at postoperative day 1, day 3, week 1, and week 3, and then 1 month later and at 3- to 4-month intervals thereafter. Intraocular pressure was measured using a noncontact tonometer or a rebound tonometer (I-care, Helsinki, Finland). An intraocular pressure of 21 mmHg or above was considered as elevated. The cornea, anterior chamber, IOL position, and VAO were evaluated by slit-lamp examination.

### Statistical analysis

In the biostatistical analysis of the study, the chi-square test was used to compare frequencies and percentages between groups. A p value of <0.05 was established as the level of significance. Because the number of cases did not fulfill the parametric conditions, to compare the mean values of the variables for the independent groups, the nonparametric Mann-Whitney U test was used. A chi-square test or a Fisher’s exact test was used to evaluate the differences between the categorical variables. Power analysis was performed according to the a priori power analysis approach using the “Mean keratometry values (D)” variable as the primary criterion. The minimum required number of subjects was calculated as 17 by accepting the probability of type 1 error (significance level) as 0.05, the power of bivariate tests as 80% (type 2 error as 0.20), and the effect size as 1.0 to compare the mean values of two different groups.

G-power software (version 3.1.9.2) was used for power analysis. The SPSS (version 21.0) software package was used for biostatistical analyses.

## RESULTS

**Figure 1:**
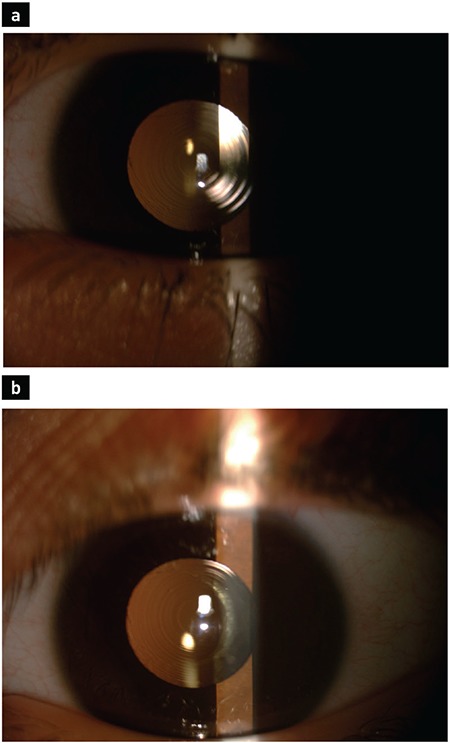
Postoperative 15^th^ month image of a 5-year-old patient who had unilateral cataract and for whom multifocal intraocular lens was implanted in the capsular bag at the end of the pediatric cataract surgery (group 1). Multifocal intraocular lens (AcrySof IQ ReSTOR) is centrally located (a). Clear optical axis can be observed (b).

**Figure 2:**
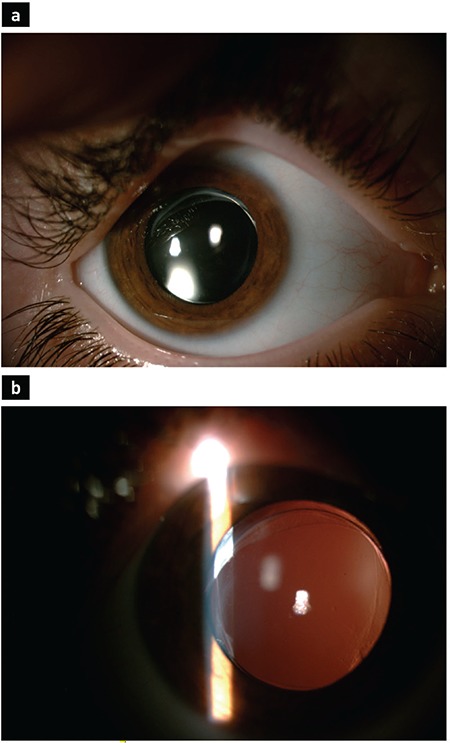
Postoperative 2^nd^ year image of a 6-year-old patient for whom a three-piece intraocular lens optic was captured behind the posterior capsulorhexis at the end of the pediatric cataract surgery (group 2). Haptics of the intraocular lens are in the bag (a), and there is optic capture through the posterior capsulorhexis (b).

This retrospective study included 52 eyes of 37 patients (12 males, 25 females; 22 unilateral, 15 bilateral). Group 1 consisted of 26 eyes of 20 patients (8 males, 12 females; 14 unilateral, 6 bilateral) and group 2 consisted of 26 eyes of 17 patients (4 males, 13 females; 8 unilateral, 9 bilateral). The mean age of the patients at the time of surgery was 8±3.4 years (range, 3-15 years) in group 1 and 7±2.4 years (range, 3-15 years) in group 2. The mean follow-up period was 28±4.7 months (range, 14-50 months) in group 1 and 30±7.4 months (range, 16-48 months) in group 2. The mean axial length was 21.88±1.02 mm (range, 19.67-23.46 mm) in group 1 and 21.48±1.1 mm (range, 19.07-23.06 mm) in group 2. The mean keratometry values were found to be 44.04±1.2 and 43.9±1.6 D in groups 1 and 2, respectively. No significant differences were observed between the groups in terms of the demographic characteristics (Table 1). Regarding the postoperative complications, uveitic reaction, posterior synechiae, and IOL decentration did not occur in either group. Glaucoma was detected in three patients in group 1 and in one patient in group 2 (p=0.298). All the patients responded to medical treatment, and no glaucoma intervention surgery was required. In the early postoperative period, cells were detected in the anterior chamber in two patients in group 1 and in three patients in group 2 (p=0.221). This reactive state was not observed on examination at postoperative week 1 (Figure 1, 2). VAO was observed in two patients in group 1 during the follow-up but was not observed in group 2 (p=0.490). There was no statistically significant difference between the groups in terms of postoperative complications (Table 2).

## DISCUSSION

Pediatric cataract surgery presents different challenges than those of adult cataract surgery. These challenges frequently include selecting a surgical technique and timing of the surgery, managing complications, and determining the dioptric power and structure of the IOL (5,6,7,8). In particular, the rate of PCO development is higher among children undergoing cataract surgery than among adults undergoing cataract surgery, and managing PCO in pediatric patients involves various additional challenges. Interventions are thus carried out to reduce the development of complications after pediatric cataract surgery. Patient’s age at the time of surgery is important as the risk of developing PCO is higher in younger age groups. Surgical technique and IOL placement are the other factors that influence PCO development (9,10). In this study, we compared two different IOL implantation techniques used in pediatric patients, i.e., those who underwent anterior vitrectomy and posterior capsulorhexis with in-the-bag IOL implantation and those in whom the IOL was captured behind the posterior capsule without an anterior vitrectomy. PCO development has been reported as a complication of pediatric cataract surgery that occurs at a rate of 100% when posterior capsulorhexis is not performed (3,4). Posterior capsulorhexis is the primary surgical intervention employed to prevent PCO development. Nevertheless, PCO and visual axis occlusion can still occur. The techniques that have been developed to prevent this are difficult to implement; they may not yield the expected results, and they may require switching to a different surgical technique than planned. Capturing the IOL behind the posterior capsulorhexis is more challenging, and the diameter of the posterior capsulorhexis is an important consideration in this technique. It should also be noted that the posterior capsule is thinner than the anterior capsule and is prone to rupture during IOL capture. In our study, occlusion of the posterior capsule opening was observed in two patients in the in-the-bag IOL group (group 1) but was not observed in the IOL capture group (group 2). Vitrectomy was not performed in the IOL capture group, though there are reports in the literature that not performing vitrectomy can increase the risk of the development of PCO. Here the anterior hyaloid membrane can provide a foundation for ongrowth of lens epithelial cells. In addition, vitreous adhesion may occur in patients who undergo vitrectomy, and retinal detachment may subsequently develop (11,12). IOL capture through a posterior capsulorhexis offers some advantages that result in better IOL fixation with less likelihood of decentration. Furthermore, strong apposition of the IOL to the posterior capsule margin prevents lens epithelial cells from advancing into the posterior capsule opening. Indeed, occlusion of the posterior capsule opening was not observed in patients with IOL capture. While IOL decentration did not occur in either group, the literature indicates that decentration is more common in patients with in-the-bag IOL implantation. The rate of IOL decentration has been reported to be 3%-20% among patients who undergo posterior capsulorhexis and anterior vitrectomy (13). Zhao et al. (14) evaluated the IOL that was placed into the ciliary sulcus and the capsular bag by ultrasound biomicropscopy and reported that IOL malposition, anterior chamber crowding, and secondary glaucoma were observed more frequently in the group for whom the IOL was fixated into the ciliary sulcus during pediatric cataract surgery in comparison to the group for whom the IOL was placed into the capsular bag. In another study, Faramarzi and Javadi (15) placed the IOL into the ciliary sulcus and the IOL was captured behind the posterior capsulorhexis in a group of pediatric cataract patients and compared them with the patients for whom the IOL was placed into the capsular bag. They found no difference between the two groups in terms of postoperative complications. Similarly, in our study, there was no difference between the in-the-bag IOL group and the IOL capture group in terms of secondary glaucoma risk and the frequency of other complications. In a similar study, Khatib et al. (16) reported no difference between the in-the-bag IOL group and the IOL capture group. Gimbel reported that IOL optic capture without performing an anterior vitrectomy after posterior capsulorhexis resulted in a clear posterior capsule opening (17,18). However, other authors have emphasized that the visual axis does not always remain clear when the optic capture is performed without vitrectomy (19). In this study, occlusion of the posterior capsule opening was not observed during the follow-up period in the group of patients who had undergone IOL capture without vitrectomy. Anterior chamber reaction was observed in the early postoperative period in two patients with in-the-bag IOL implantation (group 1) and in three patients in the IOL capture group (group 2). The fact that anterior chamber reaction occurred less frequently in the group that underwent vitrectomy may be due to the use of triamcinolone during anterior vitrectomy. Similarly, postoperative glaucoma was more common in group 1 patients who had undergone vitrectomy. The patients responded well to medical treatment, and no surgical intervention was required. In conclusion, pediatric cataract surgery differs substantially from adult cataract surgery. Numerous studies have focused on reducing the development of postoperative complications of cataract surgery in children. In pediatric cataract surgery, IOL optic capture through a posterior capsulorhexis without anterior vitrectomy was found to be slightly superior to in-the-bag IOL implantation after posterior capsulorhexis and anterior vitrectomy in terms of the development of postoperative occlusion of the visual axis. However, no statistically significant difference was found between these two techniques. Because the sample sizes of the two groups were small in our study, further studies with a larger number of patients are required to confirm the results reported herein.

## Figures and Tables

**Table 1:**
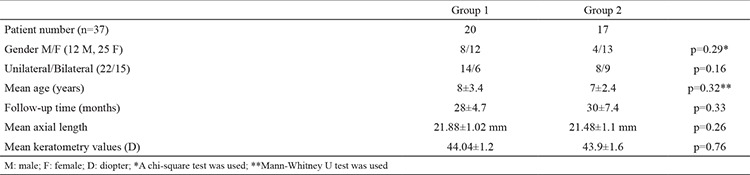
Characteristics of groups 1 and 2

**Table 2:**
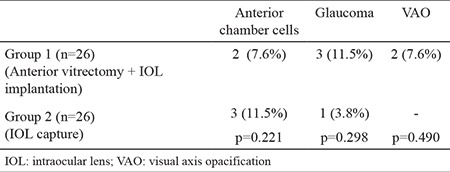
Postoperative complications in groups 1 and 2
